# Infant Directed Speech Enhances Statistical Learning in Newborn Infants: An ERP Study

**DOI:** 10.1371/journal.pone.0162177

**Published:** 2016-09-12

**Authors:** Alexis N. Bosseler, Tuomas Teinonen, Mari Tervaniemi, Minna Huotilainen

**Affiliations:** 1 Cognitive Brain Research Unit, Institute of Behavioural Sciences, University of Helsinki, Helsinki, Finland; 2 Institute for Learning and Brain Sciences, University of Washington, Seattle, Washington, United States of America; 3 Cicero Learning, University of Helsinki, Helsinki, Finland; Rutgers The State University of New Jersey, UNITED STATES

## Abstract

Statistical learning and the social contexts of language addressed to infants are hypothesized to play important roles in early language development. Previous behavioral work has found that the exaggerated prosodic contours of infant-directed speech (IDS) facilitate statistical learning in 8-month-old infants. Here we examined the neural processes involved in on-line statistical learning and investigated whether the use of IDS facilitates statistical learning in sleeping newborns. Event-related potentials (ERPs) were recorded while newborns were exposed to12 pseudo-words, six spoken with exaggerated pitch contours of IDS and six spoken without exaggerated pitch contours (ADS) in ten alternating blocks. We examined whether ERP amplitudes for *syllable position* within a pseudo-word (word-initial vs. word-medial vs. word-final, indicating statistical word learning) and *speech register* (ADS vs. IDS) would interact. The ADS and IDS registers elicited similar ERP patterns for syllable position in an early 0–100 ms component but elicited different ERP effects in both the polarity and topographical distribution at 200–400 ms and 450–650 ms. These results provide the first evidence that the exaggerated pitch contours of IDS result in differences in brain activity linked to on-line statistical learning in sleeping newborns.

## Introduction

A long-standing question in cognitive neuroscience concerns the learning processes that guide language acquisition. Infants begin life with perceptual abilities that allow them to learn any language, and their perception is shaped by experience with their native language [1–3]. Previous research indicates that both infant-directed speech (IDS) and “statistical learning” (the ability to detect the distributional and statistical patterns of phonetic units in language input) play important roles in this process, influencing both phonetic learning and early word learning [4–8]. There is also evidence that the prosodic characteristics of IDS, as compared to the less varying prosody of adult-directed speech (ADS), may promote statistical learning by enhancing infants’ attention to speech as early as 8-month of age [9]. Exaggerated pitch contours of IDS may benefit early word learning by heightening attention to the input, which in turn expedites the detection of statistical regularities (see [10] for discussion). Given that electrophysiological studies have shown newborn infants can track statistical regularities in the ambient language [7] and are sensitive to pitch variation [11], a relevant question for developmental science is whether newborn infant use both prosodic fluctuations and the statistical regularities simultaneously to learn language, or whether these cues serve distinct functions early in development.

Studies on statistical learning show that attention [12] as well as the listener’s experience with auditory input impact the rate of learning, as well as the polarity and distribution of brain responses [13–16]. In the present study, we examined the hypothesis that exaggerated pitch will affect the efficiency of computational learning in sleeping newborns and assessed the effect of both exaggerated pitch and statistical regularities on their brain responses. We specifically examined whether the pattern of brain responses to a statistical learning paradigm would vary as a function of speech register. We hypothesized that brain activity could be influenced by speech register in at least two ways.

First, differences in ERP response to ADS and IDS may be related to acoustic processing. Previous research in infants reported enhanced brain activity for IDS when compared to ADS [17–21]. A functional imaging study has shown an increase in blood flow over the frontal area of newborn brains as they listened to their mother speak in IDS, as opposed to ADS [18]. A similar increase in frontal activity to IDS has been reported using electroencephalography (EEG) power in 9-month-old infants [19]. Event-related potential studies have also revealed an enhanced response to IDS versus ADS at both the phonetic [21] and word levels [20]. We also hypothesized differences in ERP response to ADS and IDS may be related to processing efficiency. In this regard, the pattern of effects linked to statistical learning would be more broadly distributed for the ADS register as compared to the IDS register. For example, experiments with adults have shown that attention [12] as well as the listener’s experience with auditory input impacts both the rate of learning and distribution of brain responses within a statistical learning paradigm [13–16]. However, it is likely that experience with stimuli and the allocation of cognitive resources, such as attention, are linked [20, 22, 23]. In this view, processing familiar or more salient auditory input frees attentional resources for other tasks involved in language processing, such as detecting the transitional probabilities between syllables (see also [24]). Recent functional imaging results in adults support this view. Tremblay et al. [13] compared segmentation accuracy and the corresponding neural responses for segmenting speech and birdsong and found that the brain activity linked to computational learning for speech input was more focal and significantly smaller in magnitude as compared to non-speech input. A similar parallel between familiarity and the extent of brain activity has been corroborated in studies examining adult processing of native versus non-native phonemes [25–29], and the processing of known versus unknown words in young children [20, 22, 30, 31].

We were also interested in identifying the similarities in the pattern of brain responses that arise from tracking the statistics. In adults, word-initial syllables elicit both an early N100 component and a later N400 component [12, 14–16, 32–34]. The N400 has traditionally been linked to semantic expectancy [35], word category violations, or unexpected but semantically acceptable words (e.g. [36]); however, within the context of a statistical learning paradigm this response is thought to relate to the identification of recently segmented pseudo-words [14]. There is evidence suggesting the N100 response reflects cognitive processes arising from the predictive dependencies of word onset (see[34]). Whether this mechanism also contributes to newborn segmentation abilities has not been examined. The two previous electrophysiological studies investigating statistical learning in newborn infants report different patterns of brain activity [37, 38]. In the first of these studies, Teinonen et al. [38] demonstrated that exposing newborn infants to tri-syllabic pseudo-words embedded within a continuous speech stream results in a larger negative deflection to word-initial syllables of each pseudo-word compared to word-medial or word-final syllables, beginning after 300 ms. Using tri-tone pseudo-words, Kudo et al. [37] reported a broad positive deflection spanning 550 ms from stimulus onset that was significant only over frontal electrode sites. These studies differed in the type of stimuli used (speech vs. non-speech), the amount of exposure to the input the newborns received, and approach to analysis, making the relative influences of experience on brain activity linked to learning across investigations difficult to assess.

Several studies of ERP responses to native and non-native stress patterns in infants have reported a mismatch response (MMR) with a positive polarity for a non-native stress pattern [39, 40]. The positive polarity of the MMR may be dependent on the stimulus characteristics, presentation speed, or reflect an enhanced effort in processing less familiar patterns due to the involvement of weaker or less activated (immature) brain processes (see [41, 42], however, see [43, 44] for a different view). Moreover, 7-month-old infants whose brain response showed a negative deflection to a familiarized word embedded in continuous speech showed more advanced language skills at 3 years of age as compared to 7-month-old infants whose brain response showed a more distributed, positive deflection, to the same stimuli [45]. Experiments with adults have shown that attention [12] as well as the listener’s experience with auditory input impacts both the rate of learning and distribution of brain responses within a statistical learning paradigm [13–16]. However, it is likely that experience with stimuli and the allocation of cognitive resources, such as attention, are linked [20, 30, 46]. In this view, processing familiar or more salient auditory input frees attentional resources for other tasks involved in language processing, such as detecting the transitional probabilities between syllables (see also [24]). The current study asked whether evidence of sensitivity to predictive dependencies is reflected in newborn ERPs. We had two hypotheses. First, based on reports that newborns track the conditional probabilities between syllables and tones [37, 38], we hypothesized that a predictive response would be present for both the ADS and IDS. Second, based on studies showing an enhanced response for predicted input (e.g. [47, 48–50]), we hypothesized that a predictive response would manifest as an enhanced response to word medial and word final syllables for both the ADS and IDS. Infants’ and adults’ computation of statistical probabilities coincides with experimental learning research showing that both human and non-human animals are sensitive to predictive dependencies of environmental input, and that this sensitivity guides learning ([51–53], see also [54]). Although the majority of the studies on sensitivity to predictive dependencies focus on the reduction in brain activity that occurs to predictable input [55–59], in newborns, frequently presented stimuli will elicit an enhanced negative ERP within 100 ms of onset, peaking at around 50 ms [60]. A similar negative deflection has been recently reported to familiar versus unfamiliar words in 7-month-old infants [45]. Attending to the regularities in the environment is efficient in promoting learning during infancy, because probability statistics can reveal information that assists category formation across domains (see [24]).

In summary, we hypothesized that when presented with a statistical learning paradigm, ERP amplitudes as a function of *syllable position* within a pseudo-word (word-initial vs. word-medial vs. word-final, reflecting word learning) and *speech register* (ADS vs. IDS) would interact. First, a *predictive* response would occur prior to 100 ms for syllable positions with high transitional probabilities (word-medial and word-final syllables) (for review, see [61, 62, 63]), for both the IDS and ADS registers. Second, we expected an enhanced *acoustic processing* response for the IDS, but not the ADS register in the 200–400 ms measurement window (see [33]). Third, we hypothesized that differences in brain activity in response to segmented pseudo-words would be evident in the latency window linked to successful segmentation for speech in newborn infants (i.e, after 300 ms, see 38), and sensitive to the saliency of the speech register (see [13, 16]) in terms of both the topography and the polarity of the effect (see [12, 20]). For the less salient ADS register, we hypothesized that the effect of word-initial syllables would be broadly distributed across electrode sites, whereas the effect of word-initial syllables for the highly salient IDS register would occur over a small subset of electrodes, consistent with the literature on neural efficiency. In this respect, the more diffuse ERPs would presumably reflect greater cognitive effort.

## Materials and Methods

Twenty-five healthy full-term newborns were recruited at Jorvi Hospital, Espoo, Finland (11 boys, 14 girls). Of the 25 newborns, 2 were omitted from the analysis, one due to experimenter error in the recording procedure and the second due to excessive movements during the measurement. The infants were recorded 0–3 days after birth, with a mean gestational age of 40 weeks and 2.64 days (39 weeks 1 day– 42 weeks 3 days), a mean birth weight of 3,552 kg (2875–4375 kg) and a mean Apgar score of 9.47 (6–10). The study protocol was approved by the Ethics Committee for Pediatrics, Adolescent Medicine, and Psychiatry, Hospital District of Helsinki and Uusimaa, and a written informed consent was obtained from one or both parents of the newborns.

The mean number of accepted epochs for the ADS register was 587, 612, and 614 for word-initial, word-medial, and word-final syllables, respectively. The mean number of accepted epochs for the IDS register was 611, 613, and 609 for word-initial, word-medial, and word-final syllables respectively.

### Stimuli

Each speech register (ADS and IDS) consisted of 18 natural Finnish syllables, 600 ms in duration, separated by 150 ms of silence (inter-stimulus interval) throughout the entire stream. A total of 12 pseudo-words were created from the syllables, with 6 pseudo-words in each condition, and presented so that each pseudo-word was never immediately repeated, and every pseudo-word followed every other pseudo-word equally often (transitional probability from a word to any other word being 1/5), keeping the word order otherwise random. There were ten 3.55-minute-long presentation blocks consisting of this, seemingly random, stream of pseudo-words. Every other block consisted of ADS pseudo-words and the rest were IDS blocks. The order of the blocks was counterbalanced across participants. The total duration of the experiment was approximately 40 minutes.

Speech stimuli were cut from natural utterances of a female speaker recorded in an anechoic chamber [38]. Four different types of syllables were used: /k/ + vowel, /s/ + vowel, long vowel, and diphthong. The syllables were chosen so that the fundamental frequency of the voice remained relatively stable throughout the syllables. From these syllables, IDS was created by overlaying the prosodic contours excised from naturally spoken IDS registers onto the syllables using PRAAT [64].

**Fig 1:**
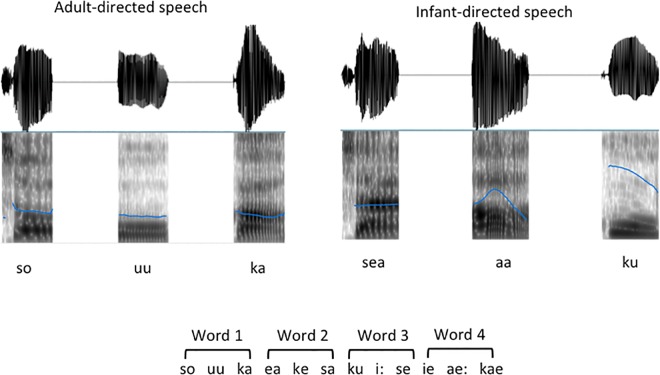
Example F0 contours, spectrograms of syllables, and schematic of the experimental procedure. Top panel. Example F0 contours and spectrograms of syllables presented in the ADS (left) and IDS (right) registers. Bottom panel. Schematic of the experimental procedure, i.e., 4 pseudo-words from the speech stream.

The ADS register average F0 was 191 Hz (range = 181–212 Hz) and the average F0 in the IDS was 212 Hz (range = 180–235 Hz). The larger range in the IDS register reflects the exaggerated pitch peaks, which reached an average of 381 Hz whereas the ADS register reached an average of 228 Hz. To make sure that pitch peaks did not mark word boundaries, we ensured that no syllables were consistently stressed for any given syllable position, and pitch peaks were distributed evenly across the syllables in words based on the fundamental frequency contours. Fig 1 shows an example of the stimuli used.

### EEG recording

The EEG was recorded in a quiet room from 8 standard electrode sites spanning the scalp. Single-use electrodes were used for recording the EEG (electrodes F3, F4, C3, C4, T3, T4, P3, and P4 according to the 10–20 system), mastoids, and EOG from the canthus and below the eye. Linked mastoids were used as a reference. Sounds were presented through two loudspeakers placed 20 cm from both sides of the infant's head. The EEG had a sampling rate of 250 Hz, and was digitally filtered offline (bandpass 0.2–20 Hz).

The EEG measurement was divided into 4 recording sessions, each approximately 10 minutes in duration. The blocks were further divided into epochs between -100 ms pre-stimulus onset to 750 ms, i.e., the duration of one syllable including the silent intervals after the syllables. After baseline correction to the pre-stimulus interval, the epochs with artifacts exceeding ±150 μV were discarded. Due to low signal-to-noise ratio (SNR), the data obtained from temporal electrode sites T3 and T4 were omitted from the statistical analysis.

Measurement windows for the 0–100 ms, 200–400 ms and 450–650 ms components were based on previous studies of statistical learning [15, 37, 38], inspection of individual averages for each newborn at each electrode site and grand averages in order to capture effects across conditions. The use of measurement windows was also a conservative choice due to variability in individual peak latencies.

### Statistical analyses

To assess the effects of exaggerated pitch on statistical learning, three separate 4-way repeated measures ANOVAs were conducted for each measurement window. Each ANOVA consisted of 4 within subject factors: speech register (ADS vs. IDS), syllable position (word-initial vs. word-medial vs. word-final), electrode site (frontal vs. central vs. parietal), and hemisphere (left vs. right) as within subject factors. The main effects and interactions for each 4-way ANOVA are presented separately for each measurement window. In our design, all prospective amplitude differences in the ERPs between the two conditions would reflect differences in learning as a function of exposure, because the stimuli were counterbalanced across speech registers and participants.

In a second set of analyses (see supplemental data), we directly compared ERP amplitudes and amplitude changes as a function of exposure. Separate statistical analyses were conducted cumulatively for each of the 3.55-minute exposure blocks for the ADS and IDS registers. This analysis allowed us to examine the contribution of each successive block to the cumulative grand averaged ERPs as a function of exposure. It also provided information on the time course of segmentation.

For each ANOVA, Greenhouse-Geisser sphericity corrections were applied when appropriate. Partial-eta-squared (η_p_^2^) was calculated for each main effect and interaction. The Bonferroni correction was applied to multiple within-subject comparisons. Post hoc tests were conducted using Tukey’s HSD method. Planned comparisons were reported as significant at the .05 level and Cohen’s *d* effect sizes were calculated using means and original standard deviations to determine the proportion of total variance attributed to each significant effect.

## Results

**Fig 2:**
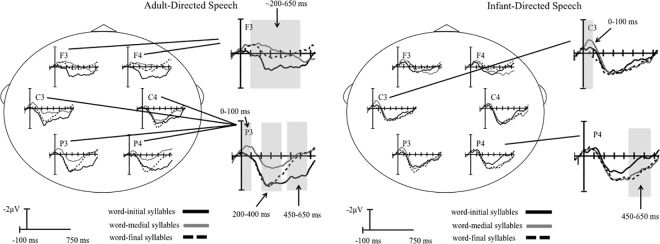
Grand-averaged ERPs for word-initial, word-medial, and word-final syllables for ADS and IDS registers collapsed across the 10 exposure blocks. Grand-averaged ERPs to the word-initial (black line), word-medial (light gray), and word-final (dashed line) syllables in the tri-syllabic pseudo-words for the ADS (top panel) and IDS (right panel) registers collapsed across the 10 exposure blocks. Infants heard each syllable 111 times. Enlarged area displays results at represented electrode sites for each measurement window. Grey bars denote significant differences in mean amplitudes between syllable positions. Negative voltages (microvolts) are plotted upward.

Fig 2 shows grand averaged ERP response to each syllable in the ADS (top panel) and IDS (bottom panel) registers collapsed across the 10 measurement blocks and all subjects. Our ERP results show clear responses in the 0–100 ms, 200–400 ms, and 450–650 ms measurement windows for the 3 syllable positions, and these responses differ for the ADS and IDS registers. Compared with the ADS register, the IDS register elicited more negative ERPs in the 0–100 ms measurement window, a larger positive response to word-medial syllables in the 200–400 ms measurement window, and larger negative responses to word-initial syllables in the 450–650 ms measurement window. For the ADS register, ERPs were dominated by a larger positive deflection spanning the entire measured response.

### 0–100 ms measurement window

**Fig 3:**
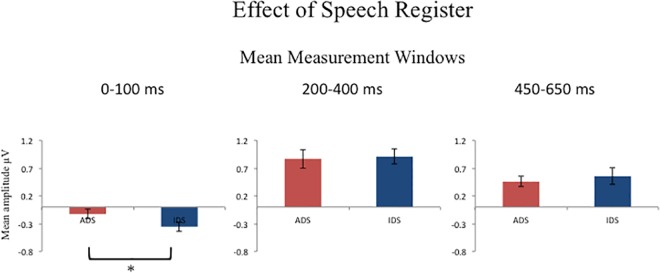
Mean amplitude for the ADS and IDS registers in the 0–100 ms, 200–400 ms and 450–650 ms measurement windows. Mean amplitude (in microvolts) for the ADS and IDS registers in the 0–100 ms (left panel), 200–400 ms (middle panel) and 450–650 ms (right panel) measurement windows averaged across the 10 exposure blocks, 3 syllable positions and 6 electrode sites. Asterisks indicate significant differences.

Fig 3 shows the overall mean amplitude for the ADS and IDS registers in the 0–100 ms measurement window.

Main effect: A four-way repeated ANOVA (2 register x 3 syllable location x 3 electrode site x 2 hemisphere) revealed a significant main effect for speech register, [*F*_(1,22)_ = 4.5, *p*<0.05, η_p_^2^ = 0.17, observed power = 0.53], reflecting larger negative mean amplitudes for the IDS (*M* = -0.354μv, *S*.*E*. = 0.085) than the ADS (*M* = -0.127μv, *S*.*E*. = 0.082) register.

Interaction: To further explore the marginally significant position x hemisphere interaction (*F*_(2,44)_ = 2.586, *p* = 0.054, η_p_^2^ = 0.124, observed power = 0.570), two 3-way (syllable position x electrode site x hemisphere) repeated measures ANOVAs for the ADS and IDS registers were conducted. These tests indicated the trend for the syllable position x hemisphere interaction was driven by the significant interaction in the IDS register [*F*_(2,44)_ = 3.548, *p*<0.05, η_p_^2^ = 0.139, observed power = 0.630]. Post-hoc tests for the IDS register indicated larger negative mean amplitudes over the left hemisphere to word-medial syllables (*M* = -0.553μv, *S*.*E*. = 0.153) than word-initial (*M* = -0.254μv, *S*.*E*. = 0.132, *p*<0.05, *d* = 0.762) and word-final syllables (*M* = -0.277μv, *S*.*E*. = 0.123, *p*<0.05, *d* = 0.642).

The syllable position x hemisphere interaction was not significant for ADS [*F*_(2,44)_ = 0.748, *p* = 0.48]; however, there was a significant main effect for hemisphere [*F*_(1,22)_ = 4.435, *p*<0.05, η_p_^2^ = 0.168, observed power = 0.521], reflecting significantly larger negative mean amplitudes over the left (*M* = -0.180μv, *S*.*E*. = 0.09) than the right (*M* = -0.046μv, *S*.*E*. = 0.098) hemisphere. No other main effects or interactions were significant.

### 200–400 ms measurement window

Fig 3 shows the overall mean amplitude for the ADS and IDS registers in the 200–400 ms measurement window.

Main effect: The same four way repeated-measures ANOVA was conducted in this time window. In contrast to the 0–100 ms measurement window, the main effect of speech register was not significant, [*F*_(1,22) =_ 0.314, *p* = 0.843]. There was a main effect of electrode site, [*F*_(2,30) =_ 15.744, *p*<0.05, η_p_^2^ = 0.417, observed power = 0.989], indicating that overall, mean amplitudes were larger over parietal (*M* = 0.99μv, *S*.*E*. = 0.168) and central (*M* = 1.095μv, *S*.*E*. = 0.143) than frontal (*M* = 0.13μv, *S*.*E*. = 0.135, frontal vs. parietal: *p*<0.05, *d* = 1.15; and frontal vs. central: *p*<0.05, *d* = 1.29) electrode sites.

Interaction: As predicted, there was a significant speech register x syllable position interaction [*F*_(2,44)_ = 3.259, *p*<0.05, η_p_^2^ = 0.129, observed power = 0.591], reflecting a significant effect of syllable position for the ADS [*F*_(2,44)_ = 5.037, *p*<0.05, η_p_^2^ = 0.186, observed power = 0.790], but not the IDS [*F*_(2,44)_ = 0.072, *p* = 0.931] register. Post-hoc tests for the ADS register indicated smaller mean amplitudes to word-medial syllables (*M* = -0.277μv, *S*.*E*. = 0.123) than to word-initial (*M* = 1.238μv, *S*.*E*. = 0.267, *p*<0.05, *d* = 0.716) and word-final syllables (*M* = 1.014μv, *S*.*E*. = 0.194, *p*<0.05, *d* = 0.620). The electrode site x hemisphere interaction was also significant [*F*_(2,44) =_ 5.002, *p*<0.05, η_p_^2^ = 0.185, observed power = 0.787], indicated larger mean amplitudes over central than parietal electrode sites in the left hemisphere (*p* = 0.011, *d* = 0.64).

### 450–650 ms measurement window

As seen in Fig 3, the 450–650 ms mean amplitude response differs in both the topographical distribution and polarity for the ADS and IDS registers.

Main effect: There were no significant main effects in this measurement window.

Interaction: As predicted, a 4-way repeated-measures ANOVA revealed a significant speech register x syllable position interaction [*F*_(2,44) =_ 7.813, *p*<0.05, η_p_^2^ = 0.262, observed power = 0.938], reflecting significant speech register effects for syllable position. To further explore this interaction, two separate 3-way (syllable position x electrode site x hemisphere) ANOVAs were conducted for the ADS and IDS registers. These tests indicated that the effect for syllable position was significant for the ADS, [*F*_(2,44) =_ 6.959, *p*<0.05, η_p_^2^ = 0.240, observed power = 0.908], but not the IDS register [*F*_(2,44) =_ 1.019, *p* = 0.369]. As shown in Fig 3, for the ADS register in the 450–650 ms measurement window, amplitudes to word-initial syllables (*M* = 1.059μv, *S*.*E*. = 0.196) were larger than word-medial (*M* = 0.138μv, *S*.*E*. = 0.197, *p* = 0.025, *d* = 0.977) and word-final (*M* = 0.174μv, *S*.*E*. = 0.166, *p* = 0.007, *d* = 1.016) syllables. Amplitudes for word-medial and word-final syllables did not differ significantly from each other (*p* = 1.00).

Paired comparisons were conducted to assess the distribution of the ERP effects for the ADS and IDS registers. These tests revealed significant speech register x syllable position interactions over the right frontal [*F*_(2,44)_ = 4.146, *p*<0.05, η_p_^2^ = 0.159, observed power = 0.702], left parietal [*F*_(2,44)_ = 3.241, *p*<0.05, η_p_^2^ = 0.128, observed power = 0.588], and right parietal [*F*_(2,44)_ = 11.681, *p<*0.05, η_p_^2^ = 0.347, observed power = 0.991] electrode sites.

As predicted, distribution of the ERP effects differed for the ADS and IDS registers, with larger positive mean amplitudes elicited to word-initial syllables over the right frontal and the left and right parietal electrode sites as compared to word-medial and word-final syllables (p<0.05, *d* = 0.71–1.05) in response to the ADS register. In contrast, for the IDS register, an effect of syllable position was observed only over the right parietal electrode site and was driven by significantly larger negative mean amplitudes for word-initial syllables as compared to word-medial and word-final syllables (p<0.05, *d* = 0.60 and 0.66, respectively).

## Discussion

The current study examined how exaggerated pitch, typical of IDS, affects the brain response in a statistical learning paradigm in newborns. We hypothesized that presenting the speech with exaggerated pitch would facilitate statistical learning in sleeping newborns, and more importantly, that the ERPs would vary as a function of two different aspects of the speech stimuli that were manipulated in the current experiment: (1) the transitional probabilities between syllables (syllable position), and (2) speech register (ADS vs. IDS). Interactions between speech register and syllable position were predicted in 3 measurement windows: at the very early 0–100 ms window, differences were expected to show an enhanced ERP for syllables occurring in predictable over unpredictable positions within pseudo-words, regardless of speech register; at the 200–400 ms window, the IDS (but not ADS) register was expected to show enhanced ERP responses to the 3 syllable positions based on the acoustic saliency of individual phonemes; and at the late 450–650 ms window, differences were expected to show patterns consistent with processing efficiency, with effects being more broadly distributed for the ADS register versus the IDS register.

Our results were consistent with these hypotheses. Event-related brain potentials differed as a function of syllable position within a pseudo-word and speech register. Our results showed that although overall ERP responses were larger in the earliest 0–100 ms measurement window for the IDS over ADS register, within each register the ERPs were larger for the syllable position with the highest transitional probability. Detecting the most predictable syllable in continuous speech may promote segmentation during infancy, because attending to the probabilistic information in language input identifies the critical elements (phonemes and words) and thus supports learning. The brain formulates predictions based on the incoming statistical regularities, allowing for more efficient processing and pattern recognition. In the current study, increasing the saliency of the input, for example by varying the prosodic contours of individual phonemes, appears to enhance statistical processing, making the response to critical features of the stimuli more robust. We suggest interpreting the early 0–100 ms effect as reflecting efficient memory trace formation for the statistical regularities that results from the same prediction based mechanism linked to predictive coding (see also [32, 38]). This early response may function to allocate cognitive resources to processing the raw statistics of the input, resulting in more efficient processing of the statistical input. The more focal effect of syllable position for IDS versus ADS pseudo-words seen in the 450–650 ms measurement window could be interpreted as more efficient processing of statistical patterns over the 40-minute exposure period during the IDS blocks. Selective attention has been shown to play a role in successful segmentation in awake adults [12, 32], and in natural learning environments IDS may be beneficial in early word learning by heightening attention to the input more generally, which in turn expedites the detection of statistical regularities.

We also observed an interaction between speech register and syllable position in the 200–400 ms measurement window (see [33]). A previous study has shown an enhanced positivity to nonnative language contrasts for 11- month old infants with a significantly larger vocabulary size at 18, 22, 25, 27 and 30 months compared to infants with smaller vocabularies [42]. Rivera-Gaxiola et al. [42] attribute the positive ERP to enhanced acoustic processing. Work with adults is consistent with this interpretation, showing an enhanced P200 response occurs with intense auditory discrimination training [65–70] or with the implantation of cochlear implants in congenitally deaf patients [71]. Other work has shown that presenting both statistical information (transitional probabilities between syllables) and the prosodic cue of increased pitch to word-initial syllables results in an enhanced positivity that peaks at approximately 225 ms (P200) for predictable, but not unpredictable, word streams in adults [33]. The authors of this later study posit that the enhanced P200 reflects enhanced auditory learning, with pitch cues functioning as attentional cues that prime language segmentation [33]. Although speculative, the significantly larger positivity in the 200–400 ms measurement window for the IDS register in this study may also be linked to auditory learning, reflecting enhanced, or more in-depth processing of the acoustic properties for the IDS register that occurs independent of their position within the pseudo-words. Importantly, our data suggest that such an enhancement may be seen even when sleeping newborns are exposed to both exaggerated pitch and statistical regularities.

Lastly, we hypothesized that the observed broader distribution of brain activity across electrode sites after 300 ms for ADS versus IDS register is linked to less successful pattern recognition, or segmentation processes. Previous work has argued that effects of syllable position in this latency window reflect the process of statistical learning with regard to word segmentation in both infants [38] and adults [15, 32, 33]. When averaged over the 10 exposure blocks, word-initial syllables elicited larger mean amplitudes for the ADS register that were broadly distributed over the right frontal and bilateral parietal electrodes. In contrast, the IDS register showed an effect of syllable position that was specific to the right parietal electrode site, and driven by more negative mean amplitudes to the word-initial syllables as compared to word-medial and final-syllables. The greater bilateral and anterior distribution of this effect for the ADS register suggests that processing statistical regularities of sounds with exaggerated pitch results in the recruitment of different brain areas than processing statistical regularities without exaggerated pitch. Research with older infants and adults have found links between processing efficiency and the distribution of the brain response, with more focal activity linked to more efficient processing for familiar and/or known words [23, 31, 46, 72]. Because infants in this experiment received equal amounts of exposure to the ADS and IDS inputs, these results cannot reflect differences in experience with the statistical regularities, but rather suggest that speech register modulates activity based on syllable position within the pseudo-word.

Our findings differ from previous studies in both the polarity and topographical distribution of effects. Using the same ADS stimuli as the current study, Teinonen and colleagues [38] reported an ERP response that differentiated between syllable positions, however, the effect was driven by a larger negative response for word-initial syllables that began after 300 ms from syllable onset. The differences between our findings and those reported elsewhere may reflect processing differences between speech and non-speech input [37], differences in baseline correction, stimulus duration, interstimulus interval, and our choice of using an alternating block design in the current study in which the speech register switched during the ERP measurement. It is possible that switching between IDS and ADS registers during testing in this study may have had an effect on processing by creating a greater cognitive load for the ADS register. Kudo et al. [37] hypothesized that the broad positive deflection observed in their results may reflect the immaturity of neural and glial cells in the newborn, leading to slower perceptual processing.

### Time course analysis: Effect of statistical input as a function of exposure

Since newborn ERPs show tremendous variability between individuals and also within an individual infant across experiments, we conducted a time-course analysis. This analysis examined the accumulated response to assess the effects of learning and stability of the response as a function of increased exposure to the statistical input. This approach allowed us to observe the temporal unfolding of ERP activity for the ADS and IDS registers (see Supplement). Our accumulative analysis revealed that the effect of syllable position for the ADS register was initially a broad positivity, similar to the effect observed by Kudo et al. [37]. However, the current study found that this initial broad positivity evolved into three distinct components with increased exposure. For the ADS register, the effect of syllable position was initially observed as a broad positivity that spanned the three measurement windows and multiple electrode sites; however, within the first 3 exposure blocks, the response evolved into three distinct components that differed in spatial distribution. In the 0–100 ms measurement window, responses to word-medial syllables elicited significantly larger negative responses over the left frontal, central and parietal electrode sites in the first exposure block. With continued exposure, this effect became attenuated at the frontal electrode site and was no longer significant when averaged over the first 5 exposure blocks. In the 200–400 ms and 450–650 ms measurement windows, the effect of syllable position differed in both polarity and distribution. Over anterior and posterior electrode sites, these effects began with larger mean amplitudes to word-initial syllables, an effect that continued over the right frontal electrode sites throughout the 10 exposure blocks, whereas effects over central and parietal electrode sites evolved into two distinct components over the course of exposure, suggesting distinct processes. Importantly, we observed that the outcomes of experience with statistical regularities differed for the ADS and IDS registers, and resulted in a ‘narrowing’ or differentiation of the ERP into distinct components with different spatial distributions. Our data, combined with the results reported in Kudo et al. [37] suggest that the initial dominant positive response is comprised of multiple generators that underlie distinct processes that become more specialized with experience.

## Conclusions

These data illustrate the speed with which the newborn brain encodes both the acoustic and statistical regularities contained in the ambient language input, thus reflecting a dynamic learning mechanism that is sensitive to input quantity and quality (see [2]). Importantly, our results show that the speech register used when addressing infants, even sleeping newborns, is an important factor in determining the patterns of brain activity, even during the earliest stages of language acquisition and provide some evidence that learning about the statistical regularities in speech is more robust when the speech is produced with exaggerated pitch contours. Our results, taken together with previous reports, are suggestive of a facilitative effect of exaggerated pitch for detecting the statistical regularities in the auditory language input that can be observed from the earliest stages of language acquisition [10].

The results from the current study lend support to the view that the same learning mechanism can yield different results, and is sensitive to both experience [13, 73] and input [74]. Our results suggest that even in a lowered arousal state i.e., during sleep, some aspect of the prosodic characteristics of IDS facilitate newborns’ access to the statistical structure of speech; however, they do not address the exact mechanism through which this occurs. One possibility is that IDS is better at attracting and sustaining infants’ processing resources as compared to ADS. There is a large body of research consistent with the view that IDS is more likely to hold infants’ attention than ADS ([75], see [76] for discussion). Follow-up studies will assess the long-term impact of IDS on language exposure. Continuing research on how attention and memory for the acoustic modifications of IDS shift as a function of language experience will advance our understanding of the interaction between the factors that guide language learning.

## Supporting Information

S1 Fig ERPs averaged cumulatively over the 10 blocks of exposure. Mean amplitudes (in microvolts) for the cumulative responses across the 10 exposure blocks for the ADS (left panel) and IDS (right panel) registers over the left and right parietal electrode sites. Grey bars denote significance differences between syllable positions (p < 0.05).
(TIF)

S1 File Time course analysis examining the cumulative responses across the 10 exposure blocks for the ADS and IDS registers. (DOCX)
