# Supplementary figures and images for: Infant Directed Speech Enhances Statistical Learning in Newborn Infants: An ERP Study
Source: PLoS One. 2016 Sep 12;11(9):e0162177. doi: 10.1371/journal.pone.0162177 (PMC5019490; doi:10.1371/journal.pone.0162177)

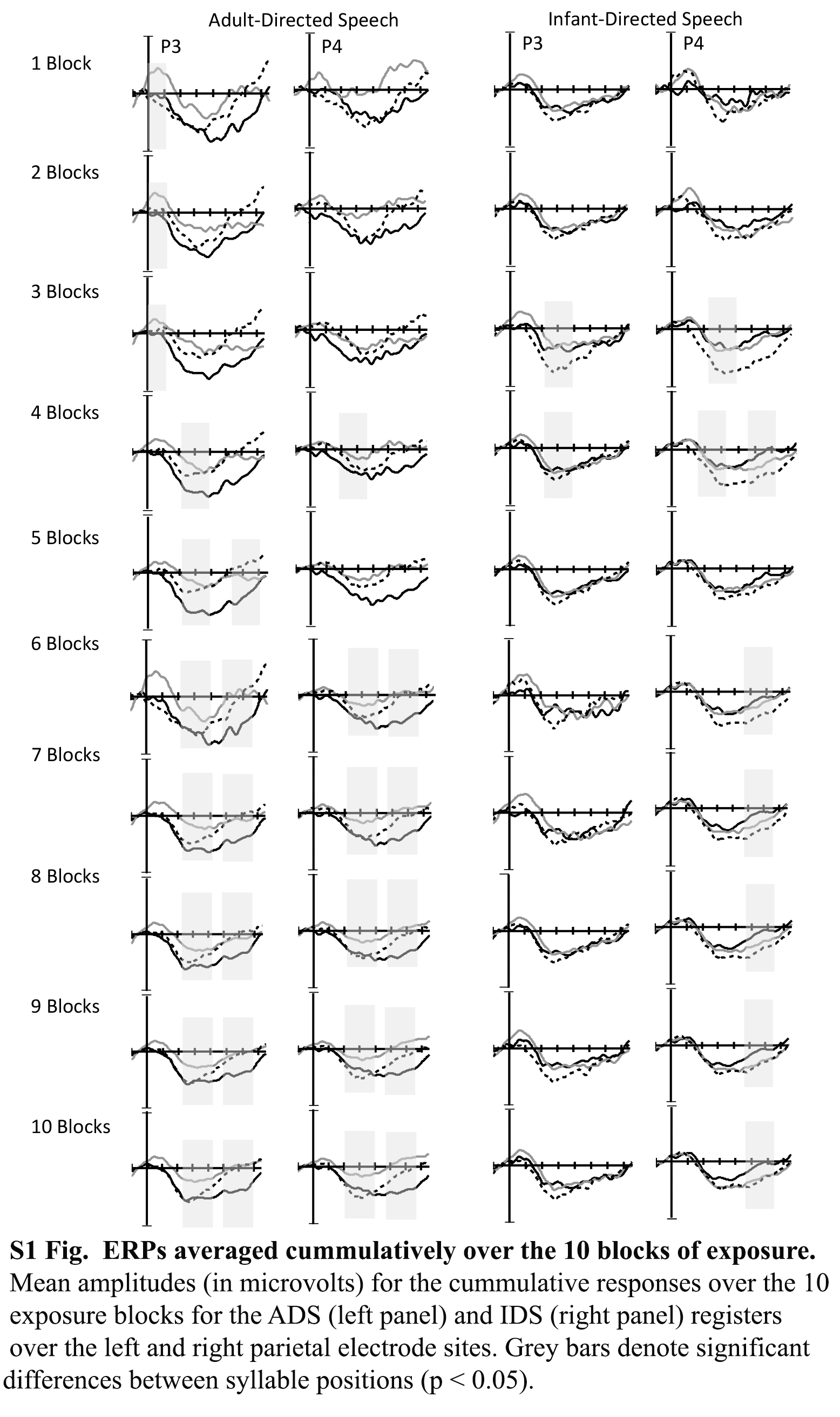

Supplement: S1 Fig — Mean amplitudes (in microvolts) for the cumulative responses across the 10 exposure blocks for the ADS (left panel) and IDS (right panel) registers over the left and right parietal electrode sites. Grey bars denote significance differences between syllable positions (p < 0.05). (TIF) [file pone.0162177.s001.tif]
