# Supplementary material for: Infant Directed Speech Enhances Statistical Learning in Newborn Infants: An ERP Study
Source: PLoS One. 2016 Sep 12;11(9):e0162177. doi: 10.1371/journal.pone.0162177 (PMC5019490; doi:10.1371/journal.pone.0162177)
Supplement: S1 File — (DOCX) [file pone.0162177.s002.docx]

**Supporting Information**

**Comparisons of cumulative blocks**

S1 Fig shows the changes in ERPs over the left and right parietal electrode sites for the ADS (left panel) and IDS registers (right panel). Our analysis revealed that the ERP changes for both the ADS and IDS registers emerged with exposure to approximately the same number of blocks, however the pattern of results that emerged differed in terms of polarity and spatial distribution.

**Comparison of cumulative blocks: Adult-directed speech**

In the 0-100 ms measurement window, the effect of syllable position was significant in the first exposure block, [*F*_(2,44)=_3.260, *p*<0.05, η_p_^2^ = 0.129, observed power = 0.591]. Post-hoc tests indicated significantly larger mean amplitudes for word-initial and word-final syllables as compared to word-medial syllables (initial vs. medial, *p*=0.010, initial vs. final, *p*=0.939, medial vs. final, *p*=0.044). The effect, however, shifted to larger mean amplitudes for word-initial syllables as compared to both word-medial and word final syllables when averaged over 1-2 and 1-3 exposure blocks (blocks 1-2, initial vs. medial, *p*=0.07, initial vs. final, *p*=0.024; blocks 1-3, initial vs. medial, *p*=0.015, initial vs. final, *p*=0.020). In addition, a significant syllable position x hemisphere interaction was observed when mean amplitudes were averaged over 1-2 exposure blocks, [*F*_(2,44)=_3.863, *p*<0.05, η_p_^2^=0.149, observed power=0.669]. This effect remained significant when averages were calculated over exposure blocks 1-4 [blocks 1-3, *F*_(2,44)=_5.586, *p*<0.05, η_p_^2^=0.203, observed power = 0.832; blocks 1-4, *F*_(2,44)=_4.182, *p*<0.05, η_p_^2^=0.160, observed power=0.706]. As indicated in our initial analysis, this interaction was driven by a larger effect of syllable position over the left hemisphere electrode sites. The effect of syllable position remained significant over the left central and left parietal electrode sites throughout the remaining exposure blocks.

Changes in ERPs in the 200-400 ms measurement window revealed two significant effects: an effect of syllable position that initially reflected differences in mean amplitudes between the word-initial versus the word-medial and final-syllables, which reached statistical significance when mean responses were averaged over the first three exposure blocks [*F*_(2,44)_=8.965, *p*<0.05, η_p_^2^ = 0.290, observed power = 0.964], and an increase in mean amplitude for word-final syllables over posterior electrode sites that was significant when averaged across the first 7 exposure blocks.

In the 450-650 ms measurement window the main effect of syllable position was significant when mean responses were averaged over the first 3 exposure blocks, [*F*_(2,44)=_5.34, *p*<0.05, η_p_^2^=0.195, observed power=0.814]. Post hoc tests indicated that mean amplitudes to the word-initial syllables were significantly larger than to word-final *(p*<0.05, *d*=0.50) syllables, but did not differ significantly from word-medial syllables (*p* = 0.499). By the sixth exposure block, the main effect of syllable position, [*F*_(2,44)=_7.102, *p*<0.05, η_p_^2^=0.244, observed power=0.914], indicated that mean amplitudes to the word-initial syllables were significantly larger than word-medial *(p*=0.02, *d*=0.800) and word-final *(p*=0.001, *d*=0.769) syllables. As shown in S1 Fig (left panel) effect of larger mean amplitudes for word-initial syllables as compared to word-medial and word final syllables was significant in the first exposure block over right frontal, the left central and the right parietal electrode sites, whereas the right parietal electrode site showed larger mean amplitudes for word-initial and word-final syllables as compared to word-medial syllables. S1 Fig (left panel) also shows that mean amplitude to word-medial syllables increased over the second and third blocks, and gradually decreased with increased exposure.

**Comparison of cumulative blocks: Infant-directed speech**

S1 Fig (right panel) shows the changes in ERP mean amplitudes for the three syllable positions in the 0-100 ms measurement window cumulatively over the 10 exposure blocks for each electrode site for the IDS register. There was a syllable x hemisphere interaction when averaged over the first 3 exposure blocks in the 0-100 ms measurement window, [*F*_(2,44)=_4.301, *p*<0.05, η_p_^2^=0.164, observed power=0.719]. As in the ADS register, post hoc tests indicated that word-medial syllables were larger in the right versus left hemisphere (*p*=0.019, *d*=0.354). The significant syllable x hemisphere interaction was observed throughout the remaining blocks. A more detailed analysis of the effect of syllable position indicated that the effect of syllable position was largest over the left central electrode site within the first exposure block until the third exposure block, with larger ERPs to word-medial as compared to word-initial and word-final syllables. This effect shifted to parietal electrode sites over the third and fourth exposure blocks.

Also within the first three exposure blocks there was a significant effect of syllable position that emerged over parietal electrode sites in the 200-400 ms measurement window. Unlike the effect for the ADS register, the IDS register elicited significantly larger mean amplitudes to word-final syllables than word-initial and word-medial syllables (initial vs. final: *p*<0.05, *d*=0.516, medial vs. final, *p*<0.05, *d*=0.490).

The effect of syllable position became significant in the 450-650 ms measurement window after four exposure blocks over the right parietal electrode site (right panel), with mean amplitudes to word final syllables significantly larger than word initial syllables (initial vs. final: *p*<0.05, *d*=0.656) and was significant again over 6 and 7 blocks (blocks 1-6, *p*<0.05, *d*=0.261 and blocks 1-7, *p*<0.05, *d*=0.261). For both 1-8 and 1-9 exposure blocks mean amplitudes for both word-medial and word-final syllables were significantly larger than word-initial (blocks 1-8 and blocks 1-9, *p*<0.05, *d*=0.100-0.235).
